# Spatial distribution of the relative risk of Zika virus disease in Colombia during the 2015–2016 epidemic from a Bayesian approach

**DOI:** 10.1002/ijgo.13048

**Published:** 2020-01-23

**Authors:** Karen Flórez‐Lozano, Edgar Navarro‐Lechuga, Humberto Llinás‐Solano, Rafael Tuesca‐Molina, Augusto Sisa‐Camargo, Marcela Mercado‐Reyes, Martha Ospina‐Martínez, Franklyn Prieto‐Alvarado, Jorge Acosta‐Reyes

**Affiliations:** ^1^ Department of Mathematics and Statistics Universidad del Norte Barranquilla Colombia; ^2^ Department of Public Health Universidad del Norte Barranquilla Colombia; ^3^ Department of Civil and Environmental Engineering Universidad del Norte Barranquilla Colombia; ^4^ Department of Public Health Research National Institute of Health Bogotá Colombia

**Keywords:** Bayes theorem, Colombia, Spatial analysis, Temporal Analysis, Zika virus

## Abstract

**Objective:**

To determine the spatial distribution of the risk of Zika virus disease in each region of Colombia during the 2015–2016 epidemic.

**Methods:**

An ecological study was designed to estimate the risks for each Colombian region using first‐order neighbors, covariate effects, and three adjacent periods of time (beginning, development, and end of the epidemic) to analyze the spatial distribution of the disease based on a Bayesian hierarchical model.

**Results:**

Spatial distribution of the estimated risks of Zika virus disease showed that it increased in a strip that crosses the central area of the country from west to east. Analysis of the three time periods showed greater risk of the disease in the central and southern zones—Arauca and Santander—where the increase in risk was four times higher during the peak phase compared with the initial phase of the outbreak.

**Conclusion:**

In the identified high‐risk areas, integrated surveillance systems for Zika virus disease and its complications must be strengthened to provide up‐to‐date and accurate epidemiological information. This information would allow those involved in policy and decision making to identify new outbreaks and risk clusters, enabling more focused and accurate measures to target at‐risk populations.

## Abstract 2

The spatial distribution of the estimated risk of Zika virus disease in Colombia was four times higher during the epidemic phase of the outbreak.

## 1 INTRODUCTION

Knowledge of the Zika virus was scarce in many high‐resource countries until an epidemic began in Brazil in 2015.^1^ A public health emergency of international importance was declared in the Americas after 48 countries had confirmed autochthonous cases of Zika by vector transmission; furthermore, the Emergency Committee of the World Health Organization (WHO) reported cases of microcephaly and other neurological disorders in disease endemic areas.^2^

In Colombia, information provided by the National Public Health Surveillance System (SIVIGILA) through the epidemiological bulletin of the Colombian National Institute of Health (NIH) reported that, since the disease was first identified in epidemiological week 29 of 2015 up to the endemic phase, close to 105 000 symptomatic cases of Zika virus infection were identified, including more than 19 000 among pregnant women. The incidence reported during the epidemic phase was of 377.7 cases per 100 000 inhabitants.^3^

Several studies have been carried out to investigate the causes and behavior of Zika virus disease in Colombia. Pacheco et al.^4^ estimated the risk of the disease in the country and found that most provinces had an accumulated incidence of between 0.1 and 129.7 per 100 000 habitants. Rojas et al.^5^ analyzed data to assess the incidence of the virus in Girardot and the island of San Andrés, and estimated attack rates of 18.43 and 12.13 per 1000 inhabitants, respectively.

Although the results of previous studies provide important information on the use of crude data, they show great variability and consider spatial relationships between observations only according to the geographical limits of each province. These studies do not consider the influence of other factors, such as settlement size, size of neighboring settlements, and the relationship between the risk area and neighboring areas, which can all affect the spatial and temporal risk of contracting the Zika virus.

Health data commonly consist of aggregated counts of disease within administrative units (small areas) such as departments and municipalities. To estimate the risk of disease, maximum likelihood estimators are typically used, such as standardized morbidity rate (SMR). These rates are variable because they depend on the expected values, which in turn depend on the size of the population.

When mapping the geographical distribution of a disease, the aim is to discover spatial patterns that help explain behavior and enable hypotheses about its etiology. The present study used Bayesian smoothing methods to estimate risk to spatially review the geographical structures of disease behavior. A wide range of models in disease mapping have been developed to offer appropriate relative risk estimates. Taking into account area information, these models provide smoothed risk maps and improve the estimates.

One of the most important studies in risk estimation is that of Besag et al.^6^ Risks are estimated using a model that captures the risk structure through incorporation of information from neighboring areas. The aim is to identify any spatial relationship that reveals the behavior of the disease, how it is distributed, and whether different factors explain this behavior. Besag et al.^6^ proposed a Bayesian hierarchical model that models risk, incorporating two random factors: one that explains the spatial dependence between neighboring areas and one that explains the residual effects. Some extensions and contributions to the Besag model are the works of Bernardinelli et al.,^7^ Best et al.,^8^ and Lawson.^9^, ^10^

The aim of the present study was to determine the spatial distribution of the risk of Zika virus disease in each region of Colombia during the 2015–2016 epidemic using a Bayesian hierarchical model.

## 2 MATERIALS AND METHODS

An ecological study was designed. The units of analysis were the administrative departments of Colombia, which is politically organized into 32 departments and four districts: Bogotá (DC), Barranquilla, Santa Marta, and Cartagena.

### 2.1 Case definition

Cases of Zika virus disease were those reported in SIVIGILA as confirmed or suspected cases. The information corresponds to the total number of cases reported by the NIH per epidemiological week (from week 32 of 2015 until week 52 of 2016) and for each department.

**Figure 1:**
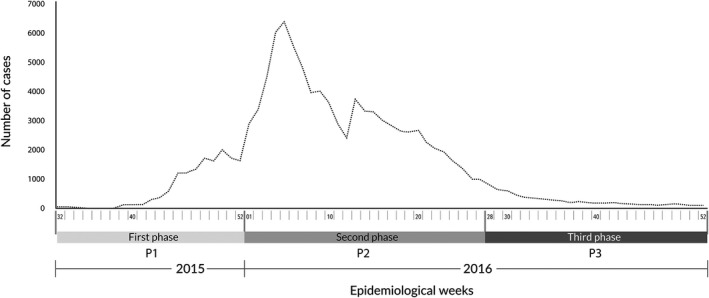
Notified cases of Zika virus in Colombia, from week 32 of 2015 until week 52 of 2016 (P1: week 32–52 of 2015; P2: week 1–28 of 2016; P3: week 29–52 of 2016).

Regarding the trend in cases of Zika virus disease over time, we evaluated the numbers of cases reported every week to SIVIGILA and defined three stages of the epidemic for analysis: (1) the first phase began with the first case reported and ended at the beginning of the epidemic's peak (P1: weeks 32–52 of 2015); (2) the second phase began at this point and ended at the beginning of a plateau in reported cases (P2: weeks 1–28 of 2016); (3) the third phase began at this plateauing and ended in the last week of 2016 (P3: week 29–52 of 2016) (Fig. 1).

### 2.2 Spatial distribution of disease risk

In the present study, an autoregressive approach to spatial mapping of the disease based on the model of Besag et al.^6^ was applied and included estimation of risk by time period (initial, peak, and endemic).

The study by Besag, better known as the BYM convolution model, is frequently used in epidemiological literature (Mollié,^11^ Ferrándiz et al.,^12^ Barceló et al.^13^). The main idea behind the model is to assume that there are risk factors that encompass more than one area of study and, consequently, the relative risks are spatially dependent. In the second level of hierarchy, the logarithm of relative risk is modeled according to spatially random effects, correlated and uncorrelated:

$$\lambda_{i}=\alpha_{0}+u_{i}+v_{i}$$

where α_0_ identifies the risk as one in the study area; *u*_i_ is the random component with spatial dependence; and *v*_i_ is uncorrelated heterogeneity. As mentioned previously, dependence is incorporated in this model based on the idea that observations from within the geographical areas will be closer to each other than observations from more distant geographical areas, thus achieving a softened estimate of the risk. Introduction of this spatial correlation structure in the model provides additional information and allows us to obtain more stable estimates of relative risks than the estimated maximum likelihood.

The relationships between risk and some bioclimatic variables have been studied; for example, altitude data taken from the Shuttle Radar Topography Mission^14^ every 3‐arc seconds (i.e. 90 m); average annual temperature identified with BIO01 data^15^ every 10‐arc minutes (i.e. 18 km); and precipitation with BIO16 data^15^ every 10‐arc minutes (i.e. 18 km).

In the present study the model was implemented through R statistical software version 3.5.1^16^ and WinBUGS 14.^17^ The QGIS 2.18.11 program^18^ was used to prepare the maps.

The project was endorsed as a risk‐free investigation by the Ethics Committees of North University and the Pan American Health Organization.

## 3 RESULTS

The main input for the implementation of the Bayesian model is the number of cases of Zika virus disease in each of the areas studied during the three time periods included in the analysis. There were 40 741 cases of Zika virus disease in total: 909 in the first phase, 37 094 in the second phase, and 2738 in the third phase. The distribution of cases for the areas studied is given in Table 1.

**Table 1:** Distribution of observed cases of Zika virus disease by stage of the epidemic (initial, peak, and endemic) by areas studied, Colombia 2015–2016

Area studied	First phase (P1‐initial)	Second phase (P2‐peak)	Third phase (P3‐endemic)	Total
Amazonas	0	205	0	205
Antioquia	87	1452	80	1619
Arauca	6	504	38	548
Atlantico	36	2053	67	2156
Bolivar	343	391	26	760
Boyaca	24	121	24	169
Caldas	9	125	28	162
Caqueta	2	721	18	741
Casanare	6	905	93	1004
Cauca	2	92	19	113
Cesar	5	888	50	943
Choco	0	27	4	31
Cordova	11	1503	17	1531
Cundinamarca	8	1838	44	1890
Guainia	0	0	1	1
Guaviare	0	24	4	28
Huila	16	3584	67	3667
La guajira	3	336	8	347
Magdalena	16	1056	19	1091
Meta	2	1553	145	1700
Nariño	8	32	10	50
Norte de Santander	10	4452	137	4599
Putumayo	22	251	15	288
Quindio	0	108	17	125
Risaralda	29	488	65	582
San Andres	201	65	9	275
Santander	9	2338	532	2879
Sucre	35	335	13	383
Tolima	19	3529	114	3662
Valle	0	8118	1.071	9189
Vaupes	0	0	1	1
Vichada	0	0	2	2
Total No. (%)	909 (2.23)	37 094 (91.05)	2738 (6.72)	40 741

**Figure 2:**
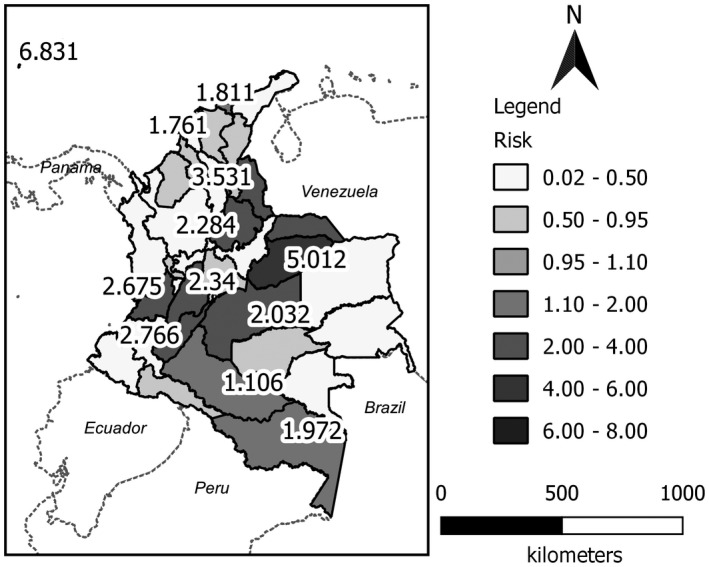
Estimated spatial risk map using the convolution model for Zika virus disease in Colombia for the 2015–2016 period.

Figure 2 depicts the studied areas, showing the spatial distribution of the estimated risks using the convolution model proposed by Besag et al.^6^ The risk in each spatially distributed area shows a trend of high values in the east, center, and south of the country. The risk structure captured shows the highest values (from 1.97 to 6.83) in these areas. The departments with the highest risk were the Archipelago of San Andrés, Providencia, and Santa Catalina (6.83); Casanare (5.01); Norte de Santander (3.53); Arauca (3.25); Huila (2.77); Valle del Cauca (2.68); Tolima (2.34); Santander (2.28); Meta (2.03); and Amazonas (1.97). In the northern zone of Colombia (the Caribbean region), it is noteworthy that the districts of Santa Marta and Barranquilla present the highest values in that region (1.811 and 1.761, respectively).

**Figure 3:**
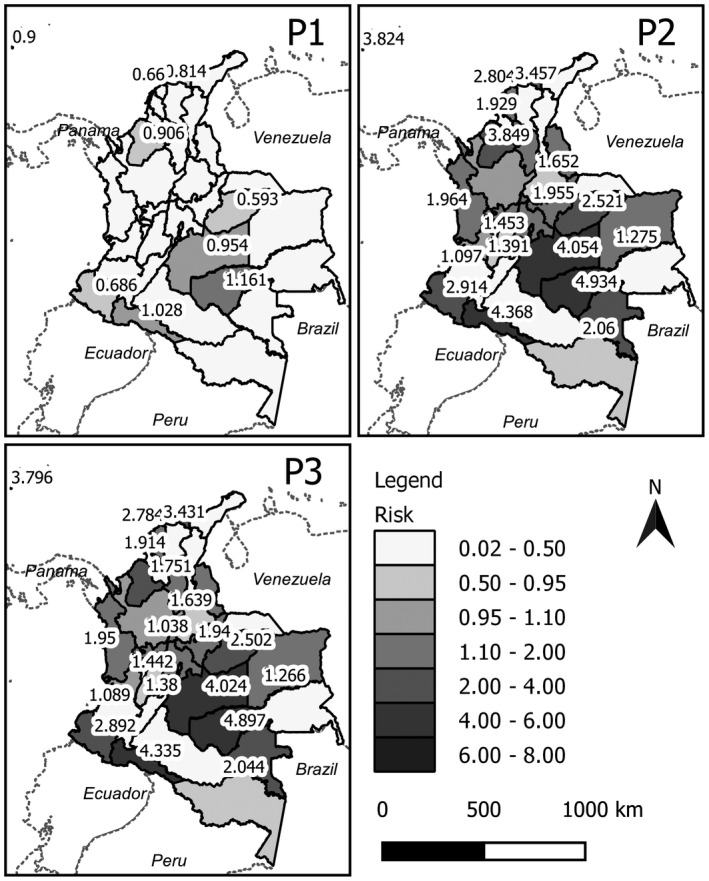
Maps of estimated spatial risk using the convolution model for Zika virus disease in Colombia by time period (P1: weeks 32–52 of 2015; P2: weeks 1–28 of 2016; P3: weeks 29–52 of 2016).

Figure 3 provides maps of the estimated risks by time period to reflect the spatial and temporal evolution of the Zika virus disease during 2015–2016. Each estimate considers the geographical and temporal relationship for the risk estimation. At the beginning of the epidemic (P1, Fig. 3), the risk values that stand out are in the departments of San Andrés, Córdoba, Atlántico, Magdalena, Casanare, Meta, Guaviare, Putumayo, and Nariño.

During the peak of the disease (P2, Fig. 3) the risk values were higher in the center and south of the country; in areas such as Casanare, Norte de Santander, Meta, and Guaviare, for example, the risk was up to four times higher than at the initial phase of the outbreak. Similar results were observed in Nariño, Putumayo, Huila, Valle del Cauca, and Tolima, among others. It was evident that the disease risk of the departments increased over time as their neighbors’ risk also increased over time. The spatial tendency observed in the second phase is related to the dynamics of the disease.

During the endemic phase of the disease (P3, Fig. 3) there was a decrease in risk in some areas, mainly in the north (Santa Marta, Barranquilla, among others), and in the center and south of the country (Meta, Guaviare, among others).

In phase 3 (weeks 29–52 of 2016), the risk values do not abruptly decrease because the risks are spatially correlated and the estimation of risk in each of the areas depends on the estimate obtained in period 2. Although the risk values do decrease in this endemic phase of the disease, the decrease is low, indicating only a small variation in the risk between phase 2 and phase 3.

**Figure 4:**
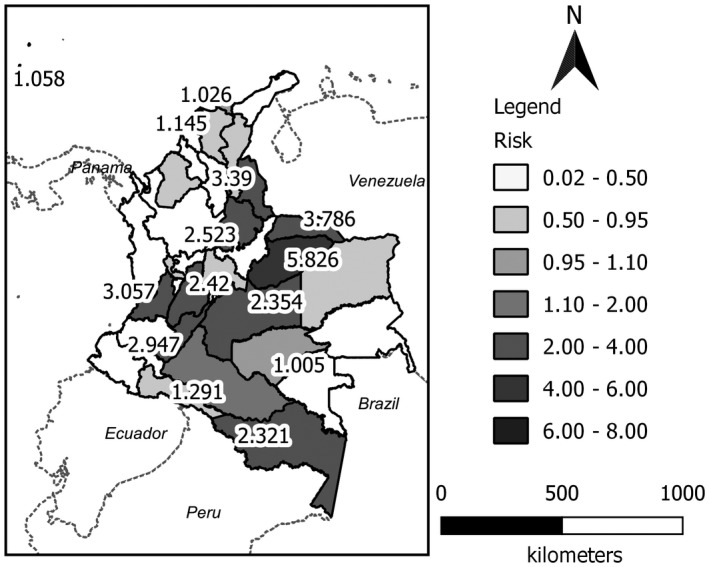
Map of the estimated spatial risk using the convolution model for Zika virus disease in Colombia, including rainfall as a covariate.

The effect of rainfall as a covariate on the risk of Zika virus disease was also analyzed. The peak period of rainfall coincided with the peak period of the disease. Results showed that areas with highest rainfall coincided with areas that had the greatest estimated risk of Zika virus disease (Fig. 4).

## 4 DISCUSSION

To our knowledge, this is the first study to explore the risk of contracting Zika virus disease in Colombia using spatial models based on a Bayesian approach. In spatial epidemiology, it is common to use the maximum likelihood estimator of the morbidity and mortality rates to estimate risk.^5^, ^19^, ^20^ These rates depend on the behavior of the expected values and work well when this value is high. The present study used smoothing methods that incorporate information on neighboring areas and covariates that allow more stable risk estimates to be obtained and that show a tendency on the map.

Our results show a spatial grouping of high risk for Zika virus disease in the departments located between the equator and the 3rd parallel north (3 degrees north of the equator), where there is a high level of rainfall during the year that might explain the space–time dynamics of the disease. Correlations between Zika virus cases and environmental factors such as daily rainfall, humidity, and average temperature have been described in studies from Brazil and China.^21^, ^22^

Inclusion of these covariates and geo‐referencing would allow an integrated surveillance system. It would generate more stable, accurate, and smoothed risk estimators to visualize trends that would improve the control of vector‐type diseases in tropical and subtropical countries.^23^ This information would allow those involved in policy and decision‐making to identify new outbreaks and risk clusters to enable more focused and accurate measures to target at‐risk populations.

The study has some limitations. Intensified surveillance was only conducted in populations at risk: pregnant women, children aged under 1 year, adults older than 60 years, and patients with comorbidities. Most data on Zika virus cases were collected through passive surveillance of people with symptoms of Zika virus and whose diagnoses were mainly clinical without laboratory confirmation; it excludes asymptomatic cases that may represent up to 40%–80% of the infected population.^24^, ^25^, ^26^, ^27^, ^28^ In addition, recent experience in Colombia in surveillance of other arboviruses (chikungunya and dengue virus) generated adequate preparation of surveillance systems; however, notification of Zika cases may have varied at different times and places.^29^ Lack of knowledge concerning disease management among public healthcare providers during the first weeks of the outbreak was corrected by the actions of the Ministry of Health and associated healthcare departments through training and awareness. Therefore, there were lower numbers of reported cases in the early stages of the outbreak (October 2015) compared with the later stages. Furthermore, it is worth noting that environmental measurements were taken at a community level and not at an individual level, which might cause an ecological bias.

From a statistical point of view, our study is based on retrospective data from a period of less than 24 months, which limits model generation due to lack of available data. This also poses a challenge to the incorporation of sociodemographic and inter‐ and intrapersonal characteristics that would help better understand other possible factors associated with Zika virus infection.

It is of great interest to review other possible models that include, for example, distance from the geo‐referenced point of a subject to a focal point, such as a healthcare center. This information would have enabled study of the dynamics of notification of symptoms and/or disease situations.

Comparing an individual's history of related diseases such as dengue and chikungunya would allow study of the probabilities of contracting Zika virus disease based on two pathologies known to be transmitted by the same vector. A risk scale for individuals could be made based on whether they had contracted any of those diseases.

We hope that these results are a starting point to continue studying the effect of covariates (for example *Aedes aegypti* pupal count, water storage containers, biological or chemical control methods, among others) on the estimation of risks in small areas. In a future study we can assess the impact of these covariates simultaneously to investigate their joint effect on the estimation of risk in each of the areas.

In conclusion, Zika virus disease is a public health emergency that, despite not having a high burden of mortality, generates neurological complications in adults and malformations in children that require prevention and control measures from healthcare authorities. Given this, risk estimation and categorization by cluster are key tools that allow the detection of high‐risk areas beyond geographical boundaries.

Although the disease's epidemic status has ended in Colombia, the results of our study identify high‐risk areas where integrated surveillance systems for Zika virus disease and its complications must be strengthened in order to provide up‐to‐date and accurate epidemiological information. This information will identify the appearance of new outbreaks and guide the response of health services.

## AUTHOR CONTRIBUTIONS

KF‐L, EN‐L, JA‐R contributed to initial study design and planning. KF‐L, HL‐S, AS‐C, RT‐M, and MM‐R contributed to data analysis; MO‐M and FP‐A contributed to data acquisition. All authors contributed to manuscript writing, revision, and approval of the final manuscript.

## CONFLICTS OF INTEREST

The authors have no conflicts of interest.
